# Mothers' and fathers' observed interaction with preschoolers: Similarities and differences in parenting behavior in a well-resourced sample

**DOI:** 10.1371/journal.pone.0221661

**Published:** 2019-08-22

**Authors:** Tine Steenhoff, Anne Tharner, Mette Skovgaard Væver

**Affiliations:** 1 Department of Psychology, University of Copenhagen, Copenhagen, Denmark; 2 Department of Clinical Child and Family Studies, Vrije Universiteit Amsterdam, Amsterdam, The Netherlands; International Telematic University Uninettuno, ITALY

## Abstract

Parenting behavior is a key factor in children’s socio-emotional development. However, little is known about similarities and differences in maternal and paternal parenting behavior, as most studies have focused on mothers. The present study investigated similarities and differences in mothers’ and fathers’ parenting behavior during observed free play with their preschool children, in a Danish well-resourced sample. We examined differences in mean scores and associations between mothers’ and fathers’ sensitivity, intrusiveness and limit-setting assessed with the Coding Interactive Behavior instrument. Additionally, we conducted confirmatory factor analysis to test the model-fit between the measurement model and parental data. Exploratory factor analysis was conducted to investigate if maternal and paternal factor structures replicated the three parenting constructs, and to explore if certain parenting behaviors seemed specifically related to either mothering or fathering. Participants included 52 mothers, 41 fathers and their 5-year old children. Similar mean scores were found for mothers and fathers on all parenting constructs. Maternal and paternal parenting behavior were not correlated. Confirmatory factor analysis revealed a poor model-fit. For both mothers and fathers, latent factors related to sensitivity, intrusiveness and limit-setting emerged, which indicated that the Coding Interactive Behavior instrument was suitable for assessment of both maternal and paternal sensitivity, intrusiveness and limit-setting. However, item loadings suggested that the instrument assessed maternal sensitivity more accurately than paternal sensitivity in our sample. Two additional factors were retrieved for fathers, i.e. paternal performance and challenging behavior, and paternal teaching behavior. This finding may suggest that additional parenting constructs need to be developed for researchers to be able to thoroughly investigate similarities and differences in mothers’ and fathers’ parenting behavior. Despite difference in factor structure, we did not identify behaviors solely related to mothering or to fathering.

## Introduction

In theories on children’s socio-emotional growth, including attachment theory [1] and theories on affect regulation [2] and socialization [3, 4], parenting behavior is considered a key factor in children’s social, emotional, behavioral and cognitive development [5–7]. Parenting behavior refers to repeated sets of specific behaviors that parents execute during interaction with their child, which can vary in intensity, frequency and duration [7]. Studies on parenting behavior have primarily focused on mothers, as fathers, for long, were not a subject of particular interest within the field of parenting research [8]. Consequently, several researchers state an urgent need of a better understanding of paternal parenting behavior [8, 9]. The importance of including fathers in studies on parenting behavior is established by recent cross-national studies, where fathers around the world, from Japan and China to The United Stated of America and Scandinavia, were found to spend more time caring for their children than previously [10–13]. Also, in Europe, equal caregiving rights for mothers and fathers has been a prominent political topic and in June 2018 the European Union countries voted in favor of a work-life balance directive, which ensures fathers two months of non-transferable paternity leave [14–17]. As fathers become increasingly involved in children’s life from early on, similarities and differences in the ways mothers and fathers parent are highly relevant to examine, as results from such studies may, eventually, broaden our understanding of how children develop and thrive.

In line with current recommendations, the number of comparative parenting studies are growing. Often, researchers examine differences in mothers’ and fathers’ sensitivity, intrusiveness and/or limit-setting, as theoretical models (e.g. attachment theory, affect regulation theory, and socialization theory) and studies, primarily conducted with mothers, have consistently linked these aspects of parenting behavior with children’s socio-emotional outcome [1–3, 18–22]. However, emerging empirical evidence suggest that from early on there seem to be a distinct pattern in the way mothers and fathers engage with their children [23, 24]. Mother-child interaction tend to involve more quiet, affectionate and socially oriented interactive patterns than father-child interaction, which is often characterized by higher levels of activity, risk taking, exploration and object oriented dyadic patterns [25–29]. Such findings have led a number of researchers to argue, that fathers might exhibit unique parenting behaviors that are relevant to identify and address alongside more traditional parenting constructs [27, 28]. Observational studies of both mother-child and father-child interactions can provide a more detailed insight into similarities and differences in mothering and fathering, and such studies might also uncover characteristic parental behaviors that have not yet been examined adequately [8]. However, studies using observational parenting measures with both mothers and fathers are still limited [8, 9, 30, 31]. Additionally, it has been questioned if today’s parenting measures assess maternal parenting behavior more adequately than paternal parenting behavior, as many assessment methods were originally developed for and used with mothers [8, 27, 32]. According to Davidov and colleagues [33] it can threaten meaningful comparisons and conclusions in case a measurement method assesses one group more accurately than another group. Thus, when fathers are included in parenting studies, it seems highly relevant to explore aspects of measurement equivalence (i.e. if a measurement method assesses different populations accurately). In a recent systematic review, Lotzin and colleagues [34] found that most observational tools for measuring parent-child interaction had not been validated on fathers. This adds to the concern that today’s parenting measures are more suited for the assessment of mothers’ parenting behavior than for the assessment of fathers’ parenting behavior.

In the current study, we aimed to investigate similarities and differences in mothers’ and fathers’ *Sensitivity*, *Intrusiveness* and *Limit-Setting* during observed interaction with their five-year-old children, in a well-resourced sample, using the Coding Interactive Behavior instrument (CIB) [35]. Also, we conducted confirmatory factor analysis (CFA) to examine the model-fit between the CIB measurement model and the participant data, and we conducted exploratory factor analysis (EFA) to explore similarities and differences in latent factor structures for mothers and fathers.

### Parental sensitivity, intrusiveness and limit-setting

Parental sensitive responsiveness is one of the most well-known and well-researched parenting behaviors, and extensive evidence show that it is one of the key factors in children’s healthy cognitive, social and emotional development [5, 6]. Sensitive responsiveness, also called sensitivity, is defined as the parent’s ability to notice the child’s signals, interpret them correctly and respond to them in a prompt and adequate manner [36]. Parental intrusiveness is antithetical to sensitive parenting behavior [37]. Whereas sensitive parenting behavior can be described as child-centered, intrusive parenting behavior often occurs when parents are unable to modulate their behavior to the child’s needs, and instead, the interaction is adult-centered, i.e. focused on the parent’s needs and wishes. Intrusive parents tend to interrupt the child’s agenda and control the dyadic interaction by e.g. attempting to engage the child in activities despite disinterest or negative reactions from the child. Often the parent acts overbearing which gives the child little or no space to adapt and respond to the parents signals. Further, intrusive parents tend to impose an immature level of functioning on the child and take care of tasks that the child is capable of handling independently, hereby restricting child autonomy [26, 38, 39]. Intrusive parenting behavior can inhibit child exploration, discourage bids for support, undermine development of independent coping-skills and elicit heightened arousal, that disrupts the child’s ability to self-regulate [37, 40]. Consequently, parental intrusiveness is considered a risk factor for healthy child socio-emotional development [37, 39].

Beyond infancy, parental sensitive discipline, which refers to parents’ ways of teaching children rules and setting limits in effective ways, becomes increasingly important for children’s social and emotional development [41]. Parental discipline is considered sensitive, when the parent takes into account the child’s perspective in situations where discipline is required, is able to maintain empathy for the child when (s)he feels frustrated and adopts child-oriented discipline methods [42]. Parental sensitive discipline is often part of an overall authoritative parenting style, where parents use so called “positive control” during interaction with their children, i.e. the parent attempts to teach, encourage and guide the child’s behavior [3, 43, 44]. Whereas parental sensitive limit-setting and monitoring of the child’s behavior have been found to promote children’s socio-emotional functioning, inconsistent or harsh discipline have consistently been linked to increased behavioral problems in children [41, 45, 46].

Often mothers have been found to act more sensitive and less intrusive than fathers during parent-child interaction [47–51]. Also, in situations that require parental monitoring and limit-setting, mothers, as compared to fathers, have been found to use more gentle guidance, i.e. direction of children’s behavior in a non-power assertive manner [52]. However, a number of studies have reported opposing results. At two different time points (i.e. 24 and 36 months), Tamis-LeMonda and colleagues [53] found mothers and fathers to act equally sensitive towards their child, and fathers scored slightly lower on intrusiveness compared to mothers. Similarly, in a study of parenting behavior Belsky and colleagues [54] reported that mothers and fathers acted equally sensitive towards their toddlers. Feldman [55] found no difference in maternal and paternal sensitivity during parents’ interaction with their infants and Feldman and Klein [56] found no mean differences in mothers’ and fathers’ warm control discipline towards their toddlers. Thus, taken together, the studies comparing mothers’ and fathers’ parenting behavior have yielded contrasting results.

In studies, where mothers were found to act more sensitive and less intrusive than fathers, and where mothers were reported to use more sensitive limit-setting compared to fathers, sensitivity and intrusiveness were assessed during free-play and limit-setting during a clean-up task [47–52]. In studies reporting opposing results, where mothers and fathers were rated equally sensitive and intrusive, and where no difference in sensitive limit-setting was identified, sensitivity and intrusiveness were also assessed during free-play and limit-setting during a clean-up task [53–56]. However, interestingly, the studies reporting opposing results differed in terms of parenting measures, as a number of different assessment instruments were used (e.g. the Emotional Availability coding system, the NICHD parenting scales, the CARE-index, and The Coding Interactive Behavior instrument). Questions have been raised regarding if and how the choice of parenting measure affects the results when comparing mothers and fathers. For example, Grossmann and colleagues [27] questioned if reported differences in sensitivity reflect true differences between mothers and fathers or whether these findings are the result of measures that were developed to assess mothers’ parenting behavior, and therefore may not adequately assess paternal parenting behavior. Grossmann and colleagues [27] also described how mothers’ and fathers’ sensitivity might be best observed in different situations and might also look slightly different, hereby challenging the assumption that mothers’ and fathers’ parenting behavior can be adequately assessed with the same instrument in the same setting. Later on, Fagan and colleagues [9] took the opposite stand and suggested moving towards a gender-neutral conceptualization, and hence assessment, of parenting. Fagan and colleagues [9] state that because parenting behaviors performed *only* by fathers have not yet been identified, the difference between maternal and paternal parenting seems to lie in the *quantity* of expressed behaviors not in *qualitatively* different behaviors. For example, rough-and-tumble play might be more characteristic for fathers, but mothers also engage in this kind of play. According to Fagan and colleagues [9] the core constructs of maternal and paternal parenting behavior–such as sensitivity and intrusiveness—are the same. In line with this perspective, Feldman [7] describes how maternal and paternal parenting behavior can differ in *content* (i.e. specific relational behaviors of each person, e.g. fathers might show more positive affect than mothers) but often the overall *form* (i.e. fundamental aspects of interaction such as sensitivity) is the same. For example, Feldman [29] found mothers and fathers to act equally sensitive towards their infant, but they differed in the behavioral way they engaged in the dyadic interaction: Father-infant interaction had a quicker tempo than mother-infant interaction, whereas mothers were rated higher on co-vocalization, affectionate touch and had longer episodes of social gaze than fathers [7, 29]. Most recently, Cabrera and colleagues [8] suggested that new parenting constructs might need to be developed, so parenting measures assess parenting practices related to both mothering and fathering, and do not only reflect behaviors characteristic for one or the other group.

### Measurement equivalence of parenting measures

Before researchers will know how to conceptualize and assess fathering, and which additional constructs that might be relevant to embrace within the field of parenting research, more knowledge is needed about similarities and differences in mothers’ and fathers’ parenting behavior [8]. Also, researchers will need to know if parental behaviors solely related to mothering and fathering exists [9]. Exploratory factor analysis is one way to examine if certain parenting behaviors seem characteristic for or uniquely related to mothers or fathers. Also, exploratory factor analysis can function as one of various ways to examine if parenting measures assess both mothers’ and fathers’ parenting behavior adequately [9]. However, very few studies have examined the measurement equivalence of available parenting measures and of the published studies, most have focused on cross-cultural, cross-ethnic or cross-language equivalence and not on gender equivalence [9, 32].

Van Leeuwen and Vermulst [57] examined the factorial validity of the Ghent Parental Behavior Scale (GPBS) for mothers and fathers. The GPBS assesses parent autonomy, discipline, positive parenting, harsh punishment, monitoring, rules, ignoring, material rewarding, and inconsistent discipline. Results showed no difference in latent factor structure for mothers and fathers, which indicated that this measure is appropriate for the assessment and comparisons of maternal and paternal parenting behavior. Finley and colleagues [58] used confirmatory factor analysis to examine the measurement equivalence of the Nurturant Fathering and Father Involvement Scales and the Nurturant Mothering and Mother Involvement Scales. Findings indicated that maternal and paternal latent parenting constructs were similar and the parenting measures therefore appropriate for assessment of both mothers and fathers. Adamsons and Buehler [32] examined measurement equivalence on three commonly used parenting constructs (i.e. acceptance, harshness, psychological intrusiveness) across mothers and fathers. The self-report measures of psychological intrusiveness and harshness demonstrated no problems with measurement equivalence across mothers and fathers. For the measure of parental acceptance the factor structure was similar for mothers and fathers, but item-level factor loadings indicated that the acceptance measure assessed mothers more accurately than fathers.

Adamsons and Buehler [32] argue that investigations of measurement equivalence should be conducted before researchers draw conclusions regarding mothering and fathering. However, studies that examine measurement equivalence across mothers and fathers are scarce [9]. Additionally, to our knowledge, most published studies have focused on measurement equivalence of parenting behavior questionnaires and not on observational measures.

### The present study

In the present study, we first investigated similarities and differences in mothers’ and fathers’ parenting behavior with the CIB coding system [35]. The CIB is designed to assess *Sensitivity*, *Intrusiveness* and *Limit-Setting* in parents and the CIB has been used in observational studies of both mothers and fathers worldwide [7, 35]. Second, we investigated if maternal parenting behavior was correlated with paternal parenting behavior. Third, we analyzed associations between mothers’ and fathers ‘parenting behavior and a number of circumstantial and contextual factors that are known to potentially affect the way parents engage with their child, i.e. educational level, leave taking, symptoms of depression and children’s behavior/temperament during dyadic interaction [59–62]. For parental leave taking, we only examined associations between this variable and paternal parenting behavior, as all mothers took leave during the child’s first year. Fourth, we conducted CFA to test the model-fit between the CIB measurement model and our participant data. The objective of CFA is to test if a hypothesized model fit the data and if the latent constructs in a sample represent the number of predefined constructs–in this case the three CIB parenting constructs. By running a CFA we aimed to examine if the CIB measurement model was suitable for the assessment of both mothers’ and fathers’ parenting behavior in our sample. Finally, we conducted EFA to investigate: 1) if maternal and paternal latent factor structures replicated the CIB parenting constructs and 2) if certain parenting behaviors seemed specifically related to mothers or to fathers. In EFA, as opposed to CFA, data is openly explored and results reveal the number of constructs needed to represent the collected data.

As described, opposing results have been reported in studies investigating similarities and differences in mothers’ and fathers’ sensitivity, intrusiveness and limit-setting. In the current study, however, we expected that mothers and fathers would obtain similar mean scores on *Sensitivity*, *Intrusiveness* and *Limit-Setting*, and that maternal parenting behavior would be positively correlated with paternal parenting behavior. We expected mothers’ and fathers’ parenting behavior to be similar and associated for several reasons. First, men and women prefer romantic partners with values, beliefs and attitudes that resemble their own [63]. Thus, we hypothesized that in the majority of families parents shared beliefs about how they wished to parent their child, which would result in similar parenting behavior across the two groups. Second, mothers and fathers continuously influence and adapt to each other’s parenting behavior [64, 65] and according to McHale [66] parents establish patterns of co-parenting, defined as the ways parents work together in their roles as parents, as early as three months following the baby’s birth. Thus, we hypothesized that mothers and fathers, even if they had entered parenthood with different attitudes towards parenting, would have established similar ways of engaging with their child, also in terms of how sensitive and intrusive they behaved and how they monitored children’s behavior through limit-setting, at 5 years old.

While the CIB is regularly used to assess fathers’ parenting behavior, we were unable to find any published literature on the validation of the CIB for fathers, and Lotzin and colleagues [34] reached the same conclusion in their systematic review. Thus, to our knowledge, the gender distribution of the sample that was used to validate the CIB is unknown. However, because the CIB is designed for the assessment of “parents”, and has been used in several comparative studies on maternal and paternal parenting behavior [7, 35], we expected that CFA would reveal a good model-fit between the CIB parenting model and our data. Also, we expected that maternal and paternal behavioral factors, retained through EFA, would replicate the three predefined CIB parenting constructs i.e. *Sensitivity*, *Intrusiveness* and *Limit-Setting*. As such, we did not expect to identify latent patterns of behavior solely related to fathering and/or mothering in our sample.

We did not develop hypotheses on the relation between parenting behavior and educational level, leave taking, depressive symptoms or child behavior, as these factors were merely included in the study as control variables.

## Method

The study was approved by The Institutional Ethical Review Board, University of Copenhagen, Department of Psychology. Approval number 2015/01. All adult participants gave written consent to participate in the study prior to assessment and parental consent was collected for all participating children as well. Data was anonymized before analysis.

### Participants

The present study is part of an ongoing longitudinal research project studying parent-child interaction and child development in a well-resourced Danish sample [67]. The participant sample consisted of 60 mothers from urban Copenhagen and their children. All children were first born. The 60 mothers were recruited for the longitudinal study in their third trimester via advertisements on social media and at local obstetricians. Women contacted the research unit if they wished to participate in a study on mother-child interaction and child development. Inclusion criteria for participation in the original study were: Primiparous, singleton pregnancy, and somatically and psychologically well. Exclusion criteria were: Alcohol or drug abuse, premature birth, physical or mental disability in the child after birth, and severe neurological or somatic disorder in the mother within the first year after giving birth [67]. Mothers and children were followed up at several time points during early childhood. When children were around five years old (*M* = 5.1, *SD* = .65), mothers, and for the first time also fathers, were invited to participate in the current study together with their child. Participants were invited for the follow-up via an e-mail describing the follow-up study and later a phone call where families could confirm whether they wished to participate or not. Two families were not invited to participate in the study, because the child was older than the target age when intake began. Six families choose not to participate, three families because they did no longer live in the country and three families because they did not have the time. The final sample consisted of 52 mothers, 41 fathers and their children. Mothers’ age ranged from 29 years to 48 years (*M* = 35.5, *SD* = 4.11) and fathers’ age ranged from 30 years to 52 years (*M* = 36.5, *SD* = 4.77). Eleven mothers participated in the study without the father. The fathers who chose not to participate in the study together with the mother mainly gave the reason that they were too busy or not interested in participating in research projects. There were no significant socio-demographic differences between the families. Overall, the sample was characterized as a well-resourced sample: Most participants had completed either a bachelor or master degree (98.1% of mothers, 87.8% of fathers) and the employment rate was high (94.2% of mothers, 97.6% of fathers). Six couples had divorced, but except from one family where the mother held sole child custody, the divorced parents shared custody over their child. 52 mothers took parental leave during the child’s first year (100%, *M* = 43.5 weeks, *SD* = 8.66) and 28 fathers took parental leave during the child’s first year (68.3%, *M* = 6.78 weeks, *SD* = 6.53) (Table 1). In Denmark, many fathers’ are granted one or two weeks of payed leave after childbirth [60]. Thus, in Table 1, fathers that are listed under “parental leave” are fathers who took at least two weeks of leave or more after childbirth. We used the Edinburgh Postnatal Depression Scale (EPDS) to screen for parental psychopathology, using the recommended cut-off score of 13 or more, to identify potential depression [68]. One mother (score of 15) and two fathers (both with a score of 13) scored above cut-off, suggesting that they may have suffered from depression at the time of assessment (Table 1). For two participants (one mother and one father) we were unable to calculate a final EPDS score, as they did not fill out the full questionnaire.

**Table 1:** Sample characteristics for mothers and fathers.

	Mothers (*N* = 52)		Fathers (*N* = 41)	
	*n*	%	*n*	%
Living arrangements				
Living together	46	88.5	40	97.6
Divorced with shared child custody	5	9.6	1	2.4
Divorced with maternal child custody	1	1.9	0	-
Years of education				
9–12 (ISCED Level 1–3)	1	1.9	5	12.2
14 (ISCED Level 4)	0	-	0	-
15 (ISCED Level 5 and 6)	19	36.5	10	24.4
17 or more (ISCED Level 7 and 8)	32	61.5	26	63.4
Occupational status				
Payed job	49	94.2	40	97.6
Stay-at-home parent	1	1.9	1	2.4
Unemployed	2	3.9	0	-
Parental leave after childbirth				
Parental leave	52	100	28	68.3
No parental leave	0	0	13	31.7
Psychopathology				
EPDS score below cut-off	50	98	38	95
EPDS score above cut-off	1	2	2	5

*Note*: ISCED = International Standard Classification of Education by UNESCO.

### Procedure

When children were five years old, parents were invited to participate in a follow-up visit in the research lab. The visit included several tasks that the child completed either alone or with one of the parents. After about one hour, children had 10 minutes of free-play together with the father, and after another hour another 10 minutes of free-play together with the mother. A standard set of age appropriate toys suitable for both boys and girls was provided, including Lego® blocks, several soft balls, a cooking set and soap bubbles kit. Toys were selected to be suitable for a variety of activities that both fathers and mothers may do at home together with their children at different levels of activation. The 10-minute periods were presented as a break for the child, and parents were instructed that they could use the time to be with their child in the assessment room and that they were free to do what they wanted to do, using the toys or not using the toys. It was explained that we wanted to observe how parents and children usually interact when they are together. After 10 minutes, the experimenter went back into the room and explained the next task, and the respective parent left the room. The whole visit was video-recorded and video material was coded afterwards.

### Measures

#### Parenting behavior

To assess mothers’ and fathers’ parenting behavior, we used the CIB instrument, preschooler version [35]. The CIB preschooler version is an extension of the original CIB coding system designed to assess parent, child and dyadic affective states and interactive styles [35]. The CIB preschooler coding system consists of 21 items for parent behavior, 16 items for child behavior, 5 items for dyadic behavior and 4 overall items applicable when the child is 3–6 years old. In the current study we used the parent-, child- and overall CIB items. Of the 21 parent items one item (Vocal Appropriateness) is only applicable when the child is between 2 and 36 months old. In the current study, children were 5 years old, and therefore the Vocal Appropriateness item was excluded from data analysis. To assess parenting behavior we coded the 20 parent items (e.g. Acknowledging, Elaborating, Criticizing, Overriding and Consistency of Style) and the 4 overall items (i.e. Child-Led, Parent-Led, Social Oriented, and Object Oriented), and to assess child behavior we coded the 16 child items (e.g. Child Positive Affect, Withdrawal, Compliance to Parent). Each behavior is coded on a 5-point scale, with 1 indicating a minimum level of the specific behavior or attitude and 5 indicating a maximum level of the specific behavior or attitude [35]. Five minutes of video-recorded parent-child interaction were coded, from minute 2 to minute 7 in the free-play session, allowing parents and children to become familiar with the laboratory setting and hereby engage in a more natural interaction, before coding began. Two experienced coders coded the interactions. One coder coded paternal interactions, while the other coder coded maternal interactions. 20 percent of the observed parent-child interactions (i.e. 9 father-child interactions and 9 mother-child interactions) were randomly picked and rated individually by both coders. Interrater reliability revealed an excellent agreement with ICC = .92.

After coding the interaction, the parent items were summarized into the three CIB parent constructs and the child items were summarized into the three CIB child constructs. The final participant score on each CIB construct was derived from the average score of all items included in that specific construct. The parent constructs are: *Sensitivity* (Acknowledging, Elaborating, Parent Gaze/Joint Attention, Positive Affect, Appropriate Range of Affect, Resourcefulness, Praising, Affectionate Touch and Supportive Presence), *Intrusiveness* (Forcing, Overriding, Negative Affect/Anger, Hostility, Anxiety and Criticizing) and *Limit-Setting* (Consistency of Style, On-Task Persistence and Appropriate Structure/Limit-Setting). The child constructs are: *Involvement* (Gaze/Joint Attention, Positive Affect, Affection to Parent, Alert, Fatigue, Initiation, Competent Use of Environment, Creative Symbolic Play), *Withdrawal* (Negative Emotionality, Withdrawal, Emotion Lability, Child Avoidance of Parent) and *Compliance* (Compliance to Parent, Reliance on Parent for Help, On-Task Persistence).

In studies where the aim is to examine maternal and paternal limit-setting a specific task where children are required to meet parental demands (e.g. a clean up task) is often included. In the present study, this was not the case. However, the CIB allows researchers to assess parental *Limit-Setting* without including a specific disciplinary task, as *Limit-Setting* in the CIB also refers to more subtle disciplining behaviors such as not allowing the child to leave the room [35]. Additionally, the *Limit-Setting* construct includes the items On-Task Persistence and Consistency of Style, why the final score on *Limit-Setting* also provides information about the parent’s monitoring of the child’s behavior and the parent’s interactive style (i.e. predictable/consistent or abrupt).

According to Feldman [7], medium-to-high test-retest reliability has been observed for the CIB constructs in samples of normative and high-risk populations and across ages, and in every sample so far, the same codes aggregated into the same overall constructs with adequate internal consistency. Also, Feldman [7] state that CFA conducted with 483 interactions showed a good model-fit, which confirmed the CIB parenting constructs. However, as previously described, the gender distribution of these 483 interactions is unknown. The CIB has been used in many different countries (e.g. France, Germany, Belgium, Italy and Brazil) and data support the instruments universal applicability [7].

### Statistical analysis

All analyses were conducted in IBM® SPSS® Statistics, version 25, except for CFA that was conducted in IBM® SPSS® Amos, version 24. First, we aimed to investigate similarities and differences in mothers’ and fathers’ *Sensitivity*, *Intrusiveness*, and *Limit-Setting*. By using independent samples t-tests we compared mean scores across the two groups. Second, we used Pearson’s correlations to test the association between maternal and paternal parenting behavior and we analyzed the internal consistency reliability of the parent composites for mothers and fathers using Cronbach’s Alpha. Third, we aimed to investigate factors that may influence parenting behavior. Thus, using one-way ANOVA, we examined the association between parental educational level and mothers’ and fathers’ parenting behavior and, using Pearson’s correlations, we tested the association between fathers’ leave taking and paternal parenting behavior, and between mothers’ and fathers’ score on the EPDS and maternal and paternal parenting behavior. Additionally, we investigated children’s mean scores on *Involvement*, *Compliance* and *Withdrawal* during interaction with mothers and fathers, and correlations between child- and parenting behavior. Fourth, we aimed to examine aspects of measurement equivalence for the CIB in our sample. We conducted CFA to test the model-fit between the CIB parenting behavior model and our data. Applied criteria to evaluate model-fit were: X^2^ (chi-square), adjusted goodness-of-fit-index (AGFI), normed fit index (NFI), comparative fit index (CFI) and root mean square error of approximation (RMSEA). Further, we conducted EFA to examine if the retained maternal and paternal factors replicated the three CIB parent constructs. EFA was conducted with Principal Axis Factoring as extraction method and Varimax with Kaiser Normalization as rotation method. Maximum Iterations for convergence was set to 25. By conducting EFA, we also aimed to explore if latent factor structures specifically related to fathering or to mothering would emerge.

## Results

### Descriptive statistics and internal consistency reliability of parenting composites

As expected, we found similar mean scores for mothers and fathers on the three parent constructs. As shown in Table 2, both mothers and fathers had high mean scores on *Sensitivity* and *Limit-Setting* and low mean scores on *Intrusiveness*. Independent samples t-tests showed no significant differences between mothers’ and fathers’ mean scores. Contrary to our initial hypothesis, none of the maternal and paternal parenting behaviors were significantly correlated. As shown in Table 2, paternal behavioral composites in general had lower internal consistency reliability than those of mothers. The internal consistency reliability ranged from moderate (*Intrusiveness* and *Limit-Setting*) to high (*Sensitivity*) for maternal behavioral composites, and from low (*Intrusiveness*) to high (*Sensitivity*) for paternal behavioral composites. If a scale is found to show low internal consistency reliability, in this case paternal *Intrusiveness*, results must be interpreted with care [69]. Thus, to further investigate if mothers and fathers acted equally intrusive or not, we used the CIB item Overriding Behavior as an index of intrusiveness in mothers and fathers, as overriding behavior is described as a main aspect of parental intrusiveness [26]. Results showed low mean scores for both mothers (*M* = 1.28, *SD* = .58) and fathers (*M* = 1.10, *SD* = .36) on overriding behavior during dyadic interaction.

**Table 2:** Mean scores and correlations between maternal and paternal parenting behavior.

	Mothers			Fathers				
	*M*	*SD*	*α*	*M*	*SD*	*α*	*r*	*P*
Parental Sensitivity	3.67	.51	.884	3.65	.33	.807	.219	.169
Parental Intrusiveness	1.13	.26	.678	1.04	.09	.155	.278	.078
Parental Limit Setting	4.68	.53	.697	4.76	.32	.611	.189	.238

*Note*: *α =* Cronbach’s alpha, *r* = Pearson’s correlation coefficient.

When analyzing factors that may influence parenting behavior we found, that educational level and paternal leave taking were not associated with parental *Sensitivity*, *Intrusiveness* or *Limit-Setting* in the current study. Both mothers and fathers had low mean scores on the EPDS (Mothers: *M* = 4.33, *SD* = 3.71, Fathers: *M* = 4.35, *SD* = 3.18). For mothers, no association was found between symptoms of depression, assessed with the EPDS, and parenting behavior. For fathers, however, symptoms of depression were positively and significantly correlated with paternal Intrusiveness (*r* = .322, *p* = .043). This finding suggest that in the current study, mothers’ parenting behavior during dyadic interaction was less affected by parental self-reported depressive symptoms, compared to fathers. Children’s behavior were similar during interactions with mothers and fathers. Children had high mean scores on *Involvement* and *Compliance* during both interactions (Mother: *Involvement*: *M* = 3.64, *SD* = .41, *Compliance*: *M* = 4.61, *SD* = .45, Father: *Involvement*: *M* = 3.70, *SD* = .27, *Compliance*: *M* = 4.81, *SD* = .23), and low mean scores on *Withdrawal* (Mother: *M* = 1.27, *SD* = .51, Father: *M* = 1.04, *SD* = .11). During both dyadic interactions, parental *Sensitivity* was positively and significantly correlated with child *Involvement*, parental *Limit-Setting* was positively and significantly correlated with child *Compliance*, and parental *Intrusiveness* was positively and significantly correlated with child *Withdrawal* (Table 3 and Table 4), suggesting similar dyadic patterns of interaction across the two groups.

**Table 3:** Correlations between maternal and child behavior measured with the CIB.

		1		2		3		4		5		6
1	Mother Sensitivity	1										
2	Mother Intrusiveness	-.58	**	1								
3	Mother Limit-Setting	.85	**	-.59	**	1						
4	Child Involvement	.76	**	-.62	**	.71	**	1				
5	Child Withdrawal	-.54	**	.49	**	-.63	**	-.70	**	1		
6	Child Compliance	.37	**	-.27	*	.48	**	.49	**	-.69	**	1

Note

** = Correlation is significant at the 0.01 level.

* = Correlation is significant at the 0.05 level.

**Table 4:** Correlations between paternal and child behavior measured with the CIB.

		1		2		3		4		5	6
1	Father Sensitivity	1									
2	Father Intrusiveness	-.38	*	1							
3	Father Limit-Setting	.55	**	-.36	*	1					
4	Child Involvement	.48	**	-.36	*	.41	**	1			
5	Child Withdrawal	-.24		.42	**	-.19		-.46	**	1	
6	Child Compliance	.18		-.17		.52	**	.51	**	-.02	1

Note

** = Correlation is significant at the 0.01 level.

* = Correlation is significant at the 0.05 level.

### Confirmatory factor analysis and model-fit

In the current study we conducted CFA for maternal and paternal data combined, as sample size recommendations for CFA [70] suggested that our sample was too small to conduct CFA for mothers and fathers separately.

The results of the CFA revealed a poor model-fit between the CIB parenting model (i.e. *Sensitivity*, *Intrusiveness*, and *Limit-Setting*) and our data on all five fit-indices: Chi-Square = 359.15, *p* = .000 (cut-off for good fit = p-value > 0.05), goodness-of-fit-index (AGFI) = .616 (cut-off for good fit = ≥ .95), normed fit index (NFI) = .679 (cut-off for good fit = ≥ .95), comparative fit index (CFI) = .765 (cut-off for good fit = ≥ .95), root mean square error of approximation (RMSEA) = .137 (cut-off for good fit = < .06) [71]. Thus, contrary to our hypothesis, CFA results indicated that the theoretical relationships hypothesized in the CIB parenting behavior model did not match our data and that the three predefined CIB parenting constructs (i.e. *Sensitivity*, *Intrusiveness*, and *Limit-Setting*) did not correspond to the latent behavioral constructs in our participant sample.

### Factor structure of mothers’ and fathers’ parenting behavior

EFA was conducted for maternal and paternal data separately, as preliminary analysis showed that both maternal and paternal data was suitable for analysis.

For mothers, exploratory factor analysis of data (*N* = 52) showed, that both Determinant (1.95), KMO criterium (.75) and Bartlett’s test of Sphericity (*Approx*. *Chi-Square* = 1145.98, *df* = .253, *p* = .000) were acceptable, indicating that the maternal data was suitable for exploratory factor analysis. For mothers, the majority of KMO-values for individual items were greater than the recommended exclusion criteria of 0.5 [72], except the following: Forcing (KMO = .12), Depressed Mood (KMO = .10), Praising (KMO = .43), Affectionate Touch (KMO = .20) and Object Oriented (KMO = .39). Because of the exploratory nature of the study, it was decided to keep items with a KMO-value below 0.5 in the dataset for further analysis of retained maternal factors. However, for mothers the item Negative Affect/Anger was excluded due to zero variation in scores (all mothers received a score of 1).

For fathers, exploratory factor analysis of paternal data (*N* = 41) also showed that Determinant (= 2.43), KMO criterium (= .67) and Bartlett’s test of Sphericity (*Approx*. *Chi-Square* = 569.85, *df* = .190, *p* = .000) were acceptable, which indicated that paternal data was suitable for exploratory factor analysis. The following items had KMO-values below 0.5: Overriding (KMO = .41), Anxiety (KMO = .39), Criticizing (KMO = .34) and Affectionate Touch (KMO = .31). As for mothers, items with a KMO-value below 0.5 was kept in the dataset for further analysis of paternal factors. However, due to zero or little variation in participant scores we had to exclude the following four items from the paternal dataset before running analysis: Forcing, Depressed Mood, Negative Affect/Anger and Child-Led.

For mothers, results showed six factors with eigenvalues above Kaiser’s criterion of 1 and together they explained 77.99% of the variance. Based on identified inflexion points on the scree-plot factor 1, 2 and 3 were retained for further analysis of maternal parenting behavior. Table 5 shows the factor loadings for the maternal sample after oblique rotation converged in 9 iterations.

**Table 5:** Factor loadings for principal axis factoring analysis with varimax rotation of mothers’ interactive behaviors (N = 52).

	Maternal factors					
CIB Scales	1	2	3	4	5	6
Acknowledging	826	-.186	.283	-.064	-.101	-.190
Elaborating	.672	.057	.204	-.010	.179	.010
Gaze/Joint attention	.375	-.102	.713	.211	.077	.183
Positive affect	.883	-.136	.069	-.187	-.034	-.212
Appropriate range of affect	.860	-.124	.247	.071	-.168	.108
Resourcefulness	.788	.153	.334	-.050	.167	-.074
Praising	.196	.372	-.103	.061	.001	-.031
Affectionate touch	.042	.162	.044	.042	.696	-.018
Supportive presence	.891	-.193	.252	-.051	-.017	-.131
Enthusiasm	.832	-.050	.427	-.096	.040	.027
Depressed Mood	-.081	-.073	-.042	-.124	.081	.439
Forcing	.000	.008	.032	.023	-.063	.194
Overriding	-.220	.863	-.031	.150	.172	.152
Negative Affect/Anger	-	-	-	-	-	-
Hostility	-.759	.321	.132	.354	-.142	-.088
Anxiety	-.169	.213	-.799	.170	-.095	-.116
Criticizing	-.468	.363	-.158	.634	.120	-.018
Consistency of style	.827	-.226	-.150	-.098	-.249	.275
On-Task Persistence	.764	-.192	.390	.152	.050	.076
Appropriate Structure/Limit Setting	.403	.287	.642	-.158	-.174	-.099
Child-led	.378	-.903	.005	-.007	-.076	-.024
Parent-led	-.479	.841	-.017	-.084	.076	-.025
Object Oriented	.047	.266	.124	.044	.072	.389
Social Oriented	.543	-.184	-.234	.320	.112	-.170

For fathers, results showed six paternal factors with eigenvalues above Kaiser’s criterion of 1 and together they explained 78.93% of the variance. Based on identified inflexion points on the scree-plot factor 1, 2, 3, 4 and 5 were retained for further analysis of paternal parenting behavior. Table 6 shows the factor loadings for the paternal sample after oblique rotation converged in 7 iterations.

**Table 6:** Factor loadings for principal axis factoring analysis with varimax rotation of fathers’ interactive behaviors (N = 41).

	Paternal factors					
CIB Scales	1	2	3	4	5	6
Acknowledging	.486	.465	.285	.302	-.099	.406
Elaborating	.169	.110	.036	.758	.076	.313
Gaze/Joint attention	.346	.709	.060	.186	-.361	-.079
Positive affect	.761	.082	.292	.246	-.240	.035
Appropriate range of affect	.612	.390	.334	.171	.018	.254
Resourcefulness	.365	.068	.110	.814	.138	-.012
Praising	.094	-.234	-.099	-.037	.905	.049
Affectionate touch	.072	.070	.039	.012	.031	.237
Supportive presence	.520	.446	.145	.520	-.034	.361
Enthusiasm	.840	-.116	.135	.263	-.078	.167
Depressed Mood	-	-	-	-	-	-
Forcing	-	-	-	-	-	-
Overriding	.026	-.859	.147	-.039	.062	-.099
Negative Affect/Anger	-	-	-	-	-	-
Hostility	-.236	-.063	-.592	-.128	-.142	-.139
Anxiety	-.188	.040	.096	.102	.653	.067
Criticizing	-.143	.030	-.744	.011	-.121	-.034
Consistency of style	.332	.119	.823	.202	-.195	-.131
On-Task Persistence	.142	.239	.244	.640	-.153	-.047
Appropriate Structure/Limit Setting	-.293	-.062	.690	.245	-.192	.452
Child-led	-	-	-	-	-	-
Parent-led	.125	-.723	-.252	-.281	.090	-.313
Object Oriented	-.164	-.289	.083	-.065	.573	-.421
Social Oriented	.552	.000	.058	.260	-.045	.543

During interpretation of the maternal and paternal factors items were interpreted as part of the factor where the highest loading occurred. Only factor loadings above .4 were considered [72]. For mothers, as shown in Table 5, the following ten behavioral items loaded positively on maternal factor 1: Acknowledging, Elaborating, Positive Affect, Appropriate Range of Affect, Resourcefulness, Supportive Presence, Enthusiasm, Consistency of Style, On-Task Persistence and Social Oriented. The following two variables loaded negatively on maternal factor 1: Hostility and Criticizing. An overlap was identified between several of the items that loaded on maternal factor 1 and the items that are included in the CIB *Sensitivity* construct. Items considered central aspects of parental sensitivity (e.g. Acknowledging, Supportive Presence and Appropriate Range of Affect) also showed high positive loadings on maternal factor 1. Taken together, this suggested that maternal factor 1 was related to sensitivity and we named it *maternal sensitivity*. For maternal factor 2, high positive loadings were identified for the items Parent-Led and Overriding, while Child-Led loaded negatively on this factor. An overlap between maternal factor 2 and the CIB *Intrusiveness* construct was identified as the item Overriding appeared both places. As overriding behavior is considered one of the main aspects of parental intrusiveness, we named this factor *maternal intrusiveness*. For maternal factor 3, the items Gaze/Joint Attention and Appropriate Structure/Limit-Setting loaded positively on this factor, while the item Anxiety showed a negative loading. The combination of the two positive loadings suggested that maternal factor 3 was related to structure and limit-setting during parent-child joint interaction. Theme wise maternal factor 3 resembled the CIB *Limit-Setting* construct and an item overlap was also identified as the item Appropriate Structure/Limit-Setting was part of both maternal factor 3 and the CIB *Limit-Setting* construct. Thus, maternal factor 3 was named *maternal structure and limit-setting*. The following items did not show loadings above .4 on any of the retained maternal factors: Praising, Affectionate Touch, Depressed Mood, Forcing and Object Oriented, which indicated that these specific aspects of parental behavior were not characteristic for the group of mothers in our sample.

For fathers, as shown in Table 6, the following six items loaded positively on paternal factor 1: Acknowledging, Positive Affect, Appropriate Range of Affect, Supportive Presence, Enthusiasm and Social Oriented. Several of the items that loaded on paternal factor 1 were the same items that are included in the CIB *Sensitivity* construct. Thus, we named this factor *paternal sensitivity*. For paternal factor 2, Gaze/Joint Attention loaded positively on this factor, while Overriding and Parent-Led showed negative loadings on this factor. In EFA, if a factor shows more negative loadings than positive loadings it often makes sense to change minus signs to plus signs and plus signs to minus signs [73], which is what we did for factor 2. Thus, paternal factor 2 was named *paternal intrusiveness*, as both Overriding and Parent-Led loaded highly on this factor. For paternal factor 3, positive loadings were found for Consistency of Style and Appropriate Structure/Limit-Setting, whereas Hostilit*y* and Criticizing loaded negatively on this factor. An overlap was identified between the items that loaded on this factor and the items included in the CIB *Limit-Setting* construct, which suggested that this factor was related to structure and limit-setting and the factor was named *paternal structure and limit-setting*. For paternal factor 4, the three items Elaborating, Resourcefulness and On-Task Persistence loaded positively on this factor. The combination of especially Elaboration and On-Task Persistence suggested that paternal factor 4 was related to teaching and we named it *paternal teaching behavior*. For paternal factor 5, the following three items showed positive loadings: Praising, Anxiety and Object Oriented. With the CIB, coders rate anxiety in case parents are silent for long periods, but also if parents for example look frequently towards the observer, show unpredictable enthusiasm and shift between emotional states [35]. The combination of Praising and Object Oriented with Anxiety–such as unpredictable enthusiasm and looks towards the cameras–suggested that paternal factor 5 might be related to goal oriented, competitive behavior, and a wish for the child and the father himself to perform during the interaction. Thus, we named the fifth paternal factor *paternal performance and challenging behavior*. For fathers, the item Affectionate Touch was the only one that did not load on any of the retained factors, which indicated that this aspect of parental behavior was not characteristic for the group of fathers in the current study.

Summing up, the findings can be described as follows: Three maternal factors were retained, which seemed to be related to *maternal sensitivity*, *maternal intrusiveness* and *maternal structure and limit-setting*. For fathers, five factors were retained and they seemed to be related to *paternal sensitivity*, *paternal intrusiveness*, paternal *structure and limit-setting*, *paternal teaching behavior* and *paternal performance and challenging behavior*.

## Discussion

The current study examined similarities and differences in observed parenting behavior in 52 Danish mothers and 41 Danish fathers during interactions with their five-year-old children in a well-resourced sample.

We found similar mean scores for mothers’ and fathers’ *Sensitivity*, *Intrusiveness* and *Limit-Setting*. Although many previous studies have reported that mothers tend to be more sensitive than fathers [47, 48], this was not the case in our study. In general, mothers and fathers showed equally “good” parenting as assessed with the CIB. If anything, fathers scored slightly lower on *Intrusiveness* and Overriding although these differences were not statistically significant. Our findings confirmed our hypothesis which stemmed from empirical knowledge related to partner preference and the importance of shared values, co-parenting patterns, and mutual parental influence on parenting style, which could mean that parents’ interactive style had become similar over the years [63–66]. Additional perspectives might add to our understanding of why mothers and fathers were found to act equally sensitive.

First of all, the study was conducted in a well-resourced sample, which could explain the high mean scores on the positive parenting behaviors for both mothers and fathers, as higher income and more years of education has been found to be predict better parenting behavior, such as more warmth and less negativity [61]. Statistical analysis showed no relation between parenting behavior and educational level. However, this may be due to low variance in data, as in samples with low variation in participant scores the association between variables tend to be weaker than in samples with higher variance [74]. Thus, the distribution of data, where most parents were rated high on *Sensitivity* and *Limit-Setting* and low on *Intrusiveness* and most had finished a bachelor or master degree, could explain why we failed to identify a relation between educational level and parenting behavior. Also, close to 70 percent of the fathers took parental leave, which suggest that the fathers who participated in the current study were in general highly involved in their children’s life from early on. In a cross-national analysis, including data on fathers from Denmark, Australia, the United Kingdom and the United States, Huerta and colleagues [60] found that fathers who took two or more weeks of leave were more engaged in child care activities (e.g. giving a bath and getting the child to bed). Fathers who took leave around childbirth were also more involved with their children at 2–3 years than fathers who did not take leave [60]. Fathers’ involvement in childcare can facilitate father-child bonding [75] and Feldman [55] found that fathers who took part in caregiving activities were more sensitive during dyadic interaction. Thus, it is possible, that most fathers were rated high on *Sensitivity*, because they knew their children well and were able to understand and respond appropriate to their children’s signals, as a result of consistent involvement in children’s life from early on. However, similar to our results on relations between educational level and parenting behavior, statistical analysis showed no association between paternal leave taking and fathers’ parenting behavior. Again, this finding could be due to low variance in sample data. It may also be, that fathers who took leave and fathers who did not take leave were equally involved in their children’s life, which could explain the lack of correlation between leave taking and paternal parenting behavior. For example, Yeung and colleagues [76] reported that better educated fathers, compared to less educated fathers, tend to spend more time with their children because they are more concerned with their children’s development. Such findings suggest, that fathers from well-resourced backgrounds in general tend to practice involved fatherhood. Also, results from studies conducted with Scandinavian families found that fathers’ caregiving involvement is strongly associated with maternal employment [10]. For example, in families where mothers worked full time, almost 50 percent of the times children were given a bath, it was the father who did it [77]. In the current study, data suggested that mothers were career oriented and driven as, except for one, all had bachelor degrees and more than half had master degrees, and some had even obtained PhDs. Also, 94.2 percent of the mothers were employed. Thus, similar mean scores on *Sensitivity* for mothers and fathers could be a result of a dual-caregiver family model, where both parents were actively involved in caregiving from when mothers finished maternity leave, and possible prior to that. Scandinavian countries are known for promoting father involvement and the dual-earner/dual-caregiver family model [10]. It seems likely that the nationality of the participants may also contribute to our understanding of why mothers and fathers showed equally good parenting behavior, as political discourse and facilities, such as father-child play centers, continuously encourage Danish fathers to spend time with and engage in their children’s life. Additionally, descriptive statistics showed that only three parents reported symptoms of depression, with scores equal to or just above cut-off, and that children, during interaction with both mothers and fathers, had high mean scores on *Involvement* and *Compliance*, and low mean scores on *Withdrawal*. Empirical evidence have linked both parental psychopathology and children’s challenging behavior and/or difficult temperament with lower levels of parental sensitivity [59, 62]. Thus, the low-risk, non-clinical, characteristics of the sample and children’s involved and compliant behavior are also likely to have had a positive influence on maternal and paternal parenting behavior results. Also, overall, correlations between parent and child behavior revealed similar dyadic patterns for mothers and fathers, with positive associations between *Sensitivity* and child *Involvement*, *Limit-Setting* and child *Compliance*, and *Intrusiveness* and child *Withdrawal*.

Another perspective, which might also contribute to our understanding of mothers’ and fathers’ similar mean scores on all parent constructs, is the research setting. Lamb and Lewis [65] describe how different research settings impose different constrains on parents and how most researchers do not sample context in a way that allow different parental styles to be expressed. For example, it has been argued that more traditional set-ups to observe parenting behavior have favored mothers, whose parenting behavior usually involves more quiet, warm and socially oriented interactive patterns whereas fathers’ parenting behavior is more focused on activation and often centers around exploration [8, 24, 27, 28]. Thus, it might be that mothers and fathers can be equally sensitive, but differ in terms of situations where their sensitive parenting behavior is best observed. In the free-play situation no specific instructions were given and parents were encouraged to interact with their children as they wished. Also, different types of toys were provided for both quiet and social play and for more activating or teaching play. This may have allowed for naturalistic maternal and paternal parenting behavior to evolve without restricting maternal or paternal styles, which could explain why mothers and fathers were rated equally sensitive. In addition, as previously described, questions have been raised regarding the consequences of assessing fathers’ parenting behavior with coding systems that have been developed for and primarily used with mothers, i.e. if such measures lead to fathers being reported less sensitive than they really are [27]. The CIB coding system, however, was designed to assess parents [35], and studies using the CIB consistently report similar scores on the parenting constructs for mothers and fathers, including *Sensitivity* [29, 55, 56]. Thus, it is possible that the CIB captures both maternal and paternal sensitive parenting behavior adequately during free play, even if fathers’ and mothers’ sensitivity might look slightly different and might be best observed in different situations, as Grossmann and colleagues [27] proposed.

Contrary to our hypothesis, mothers’ and fathers’ parenting behaviors were not correlated, despite similar mean scores across groups. While this finding is most likely due to low variation in scores, additional factors, that we did not examine, can also be relevant to consider when parents’ behavior are found to be uncorrelated, as they may have had a mediating effect on parent’s interactive style. For example, studies show that parenting stress and relationship satisfaction can affect how parents interact with their child on day-to-day basis and fathers have been reported to be particular sensitive to the spillover effect, which is the transmission of mood and behavior from one setting to another [65, 78, 79]. In line with this perspective, results suggested that self-reported depressive symptoms had a greater impact on fathers’ parenting behavior in the current study, as depressive symptoms were positively associated with *Intrusiveness* for fathers, whereas no associations were found between EPDS score and mothers’ parenting behavior. While parents in general reported low levels of depressive symptoms, and were rated high on *Sensitivity* and low on *Intrusiveness*, it is possible mothers’ and fathers’ parenting behaviors were uncorrelated because parents adjusted to their co-parents’ state of mind during the time of assessment. For example, in a study by Schoppe-Sullivan and colleagues [80] fathers were found to act more sensitive towards sons with an insecure relationship with mothers than towards sons with a secure relationship with mothers, which indicates that one parent can be motivated to act more sensitive when the child’s relation to the other parent is less optimal. Thus, parents’ do not only adapt to each others interactive style by modelling the co-parents behavior, but also sometimes by compensating for the partners lack of e.g. sensitivity.

For the CFA, results revealed a poor model-fit between the participant data and the CIB parenting model. This finding suggested that the CIB did not measure maternal and paternal parenting behavior adequately in our study. However, due to a small sample size we were not able to investigate if the model-fit was poor for one group and not for another, or if the CIB model was a poor fit for both mothers’ and fathers’ parenting behavior. Thus, while we suggest that mothers’ and fathers’ similar mean scores could be the result of a measurement method designed for both mothers and fathers, that assessed maternal and paternal parenting adequately, our results from the CFA initially indicated the opposite. However, EFA results showed similar factors for mothers and fathers (i.e. *sensitivity*, *intrusiveness* and, *structure and limit-setting*), and the items that loaded on these maternal and paternal factors overlapped with items included in the three corresponding CIB parent constructs (i.e. *Sensitivity*, *Intrusiveness* and *Limit-Setting*). This finding suggest that the CIB measurement method was indeed appropriate for the assessment of both mothers’ and fathers’ *Sensitivity*, *Intrusiveness* and *Limit-Setting* in our sample. Yet, further inspections of maternal and paternal factors showed that the *maternal sensitivity* factor, compared to the *paternal sensitivity* factor, to a greater extent replicated the CIB *Sensitivity* construct. The items Elaboration and Resourcefulness, that are included in the CIB *Sensitivity* construct, showed high loadings on the *maternal sensitivity* factor, but weak loadings, below .4, on the *paternal sensitivity* factor. This finding suggest that maternal data matched the theoretical relationships hypothesized in the CIB sensitivity model better than paternal data, which indicate that the CIB instrument measured mothers’ sensitivity more accurately than fathers’ sensitivity in the current study. For fathers, Elaboration and Resourcefulness instead loaded highly on the *paternal teaching* factor, which show that these aspects of behavior were also observed during father-child interaction. However, as Elaboration and Resourcefulness, together with the item On-Task-Persistence, formed an independent paternal factor, it is possible that for mothers, these parental behaviors were part of an overall sensitive parenting style, whereas for fathers these aspects of parenting were best observed during specific types of father-child interactions. Such findings highlight the importance of both appropriate parenting measures and research settings in comparative studies, and reflections in terms of how the choice of method may or may not have influenced results. For example, if ratings on Elaboration and Resourcefulness are included in the final *Sensitivity* score, mothers may receive higher scores than fathers, in case the research setting do not facilitate interactions where these aspects of paternal parenting behavior fully unfold.

As two additional behavioral factors were identified for fathers (i.e. *paternal teaching behavior* and *paternal performance and challenging behavior*), it seems likely that the poor CFA model-fit was related to fathers’ parenting behavior in our sample. The number of factors retrieved for fathers, and the relationship between behavioral items, did not correspond to the number of constructs, and the item structure, in the theoretical CIB model, which could be the main reason behind the poor model-fit. However, overall, the CIB assessed both paternal sensitivity, intrusiveness and limit-setting, but the identification of two additional paternal factors indicated, that the CIB did not capture all aspects of fathers’ parenting behavior in the current study. A number of researchers have described teaching, goal-oriented tasks, competitive and challenging behaviour as typical elements in father-child interactions, why the identification of the two additional paternal factor structures might come as no surprise [8, 24, 27, 28, 81]. However, in the literature these aspects of paternal behavior are often subscribed to content level instead of construct level, i.e. referred to as parenting behaviors, more common for fathers than for mothers, as opposed to core aspects of parenting behavior in line with for example sensitivity and intrusiveness. The identification of these paternal behaviors as underlying factor structures in our group of fathers might suggest that *paternal teaching behavior* and *paternal performance and challenging behavior*, are not just elements in fathers’ parenting behavior, but instead fundamental underlying aspects of fathering. As previously described, Cabrera and colleagues [8] argue that studies including both mothers and fathers might lead to the identification of new parenting constructs. According to Cabrera and colleagues [8], assessment of parenting constructs that capture both mothering and fathering are vital when researchers aim to determine how mothers and fathers are similar, different, complementary, and additive. In line with this perspective, and based on the findings of the current study, parental behaviors related to *teaching*, and to *performance and challenging behavior*, might be relevant to address in future parenting studies next to more traditional constructs such as *Sensitivity*, *Intrusiveness* and *Limit-Setting*.

Further inspection of factors showed than none of the retrieved maternal or paternal factors fully replicated the item structure of the three CIB constructs. According to Feldman [7] each CIB construct includes items that are considered to be central for this construct at any age, i.e. Acknowledging is a fundamental part of parental sensitivity, while other items can differ across cultures and depend on child age. Thus, cultural factors and factors related to child age might explain why some items did not load on any of the retrieved factors. For example, the item Affectionate Touch is included in the CIB *Sensitivity* construct, but this item did not load on the maternal sensitivity factor or on any of the other retained maternal factors. Nor did this item load on any of the factors retained for fathers. This indicates, that mothers and fathers in the current study did not often touch their children affectionately during the free play. Northern European countries have been described as low-touching countries as compared to for example Southern European countries where individuals tend to touch each other more often and have smaller personal spaces [82, 83]. Thus, in a Danish sample, affectionate touch might not be as characteristic for sensitive parenting behavior as in samples from other European countries or as in Israel, where the CIB is developed [35]. Also, according to Ferber and colleagues [84] parents have been found to touch their children less frequently as they age. Thus, it is possible that parents in the current study would have touched their children more had the children been younger.

We found that the majority of behavioral items loaded on both maternal and paternal factors, despite difference in factor structure and number of retained factors across the two groups. This finding indicated that the specific behaviors that each CIB item is related to (e.g. Elaborating, On-Task Persistence, and Supportive Presence) were performed by both mothers and fathers. Thus, we did not identify parenting behaviors solely related to mothering or to fathering in our sample. However, the two additional paternal factors indicated, that parental behaviors that centered around teaching, and performance and challenging behavior, were more characteristic for father-child interactions as compared to mother-child interactions. Only two of the items included in analysis showed loadings above .4 for one group but not for the other. These items were Praising and Object Focus and they loaded on the *paternal performance and challenging behavior* factor, but not on any of the maternal factors. A potential explanation of this finding might be, that fathers were include in the longitudinal study for the first time and mothers had attended several assessments in the past. Thus, it is possible that praising and object focus were more frequently observed during father-child interactions because fathers were unfamiliar with the situation and felt more obliged to perform than mothers, who were used to the research setting. This hypothesis seems supported by the fact that the item Anxiety also loaded highly on the paternal *performance and challenging behavior* factor. In this light, it is possible that the paternal *performance and challenging behavior* factor could be a result of different enrollment points for mothers and fathers more than a result of general differences in mothers’ and fathers’ parenting behavior. Also, in the current study, fathers participated in free-play with children before mothers. Thus, the order of dyadic interactions may have impacted results. For example, it is possible that parental behaviors related to teaching, challenge and performance, were more characteristics for fathers as a result of being the first parent to interact with their child, at a point where children might have had had more energy, than during interaction with mothers.

However, while different circumstantial factors may have impacted results, the identification of *teaching behavior* and *performance and challenging behavior* as characteristic for fathers are in line with current theoretical assumptions and emerging empirical evidence on differences in mothering and fathering. Also, according to Paquette [28] these aspects of fathering play an important role in children’s socio-emotional development, as fathers’ tendency to challenge their children and encourage risk-taking and exploration, empower children and improve their ability to open up and meet the demands of the outside world. In this light, it is important to note that the study was conducted with preschool children. Thus, *paternal teaching*, *performance* and *challenging behavior* could be characteristic for fathering at this specific point in children’s life in particularly. Preschool years mark the beginning of a transformational period, where children are soon to be enrolled in school, and skills related to self-control, cooperation, communication and motivation to learn, are important for children to be able meet the new environmental demands [85]. Also, during this stage, children develop new social skills, such as turn-taking and the ability to listen to others point of views and respond appropriately [85]. As such, the identification of *teaching*, *performance and challenging behavior* may be interpreted as fathers’ way of meeting children’s increasing need for activation and stimulation at this point, but also as fathers’ (potentially unique) way of teaching children how to adapt to a world of social and emotional demands.

### Limitations and future directions

The findings of the current study should be interpreted with reference to a number of limitations.

For fathers, the reliability of the *Intrusiveness* composite was low why paternal *Intrusiveness* must be interpreted with care. However, similar mean scores were also identified for mothers and fathers on Overriding behavior. We examined associations between mothers’ and fathers’ parenting behavior and a number of factors that are known to potentially influence dyadic interaction, but additional factors, such as parenting stress and relationship satisfaction were not assessed in the current study, even though they may have affected results. Also, we did not investigate how child gender potentially influenced mothers’ and fathers’ parenting behavior, even though empirical evidence suggest that mothers and fathers may interact differently with boys and girls [86].

Because of a small sample size we were unable to conduct CFA for mothers and fathers separately. Results revealed a poor fit between our data and the CIB parent model and while we suggest that the poor model-fit was caused by a lack of correspondence between the paternal data and the CIB parent model, we can not be sure that the poor model-fit was indeed related to fathers. To answer this question, the study would need to be replicated with a participant group big enough for CFA to be conducted separately for the two groups. We did not identify behaviors solely related to mothering or to fathering, but EFA results revealed certain behaviors more characteristic for fathers than for mothers in the current study. However, to be sure that these parenting behaviors are in fact more characteristic for fathers, EFA would need to be replicated in studies with bigger sample sizes and with participants from diverse socio-economic backgrounds. Also, EFA would need to be replicated in studies where mother-child and father-child interactions are randomized.

Additionally, Fagan and colleagues [9] argue, that to evaluate if differences in mothers’ and fathers’ parenting behavior are of significant importance, which could mean that mothering and fathering should be conceptualized differently or that additional parenting constructs should be included in parenting measures, the association between these behaviors and child development should be investigated. In the current study, we did not investigate associations between *teaching behavior* and *performance and challenging behavior* and child development. Thus, while we were able to identify that mothers’ and fathers’ differed in their overall interactive style, we do not know if and how parental *teaching behavior* and *performance and challenging behavior* potentially affected children’s socio-emotional development. Neither do we know if mothers’ and fathers’ *teaching behavior* and *performance and challenging behavior* affected children’s development in similar or different ways. Thus, to be able to draw substantial conclusions regarding how important the identified differences in maternal and paternal parenting are, it would be highly relevant to examine the potential effect on child development. In line with this perspective Cabrera and colleagues [8] recommend, that future parenting studies should not only examine differences and similarities in mothers’ and fathers’ parenting behavior, but also investigate how each parent individually and together contributes to child development. As the study was conducted with preschool children, the potential effect of *teaching*, *performance* and *challenging* behavior during this transformational and vulnerable developmental stage in children’s life seems particularly important to address in future studies.
